# Dietary restriction promote sperm remodeling in aged roosters based on transcriptome analysis

**DOI:** 10.1186/s12864-024-10544-3

**Published:** 2024-07-08

**Authors:** Wenjie Liang, Yuehua He, Tingqi Zhu, Binbin Zhang, Shuangxing Liu, Haishan Guo, Pingquan Liu, Huayuan Liu, Donghua Li, Xiangtao Kang, Wenting Li, Guirong Sun

**Affiliations:** 1 The Shennong Laboratory, Zhengzhou, 450002 China; 2 College of Animal Science and Technology, Henan Agricultural University, Ping’an Avenue 218#, Zhengdong New District, Zhengzhou, 450046 P. R. China; 3 Henan Fengyuan Poultry Co., Ltd, Nanyang, 473000 China

**Keywords:** Molting, Roosters, Reproductive performance, Spermatogenesis, Transcriptome.

## Abstract

**Background:**

The breeder rooster has played a pivotal role in poultry production by providing high-quality semen. Typically, fertility peaks between 30 and 40 weeks of age and then declines rapidly from 45 to 55 weeks of age. Research into improving fertility in aging roosters is essential to extend their productive life. While progress has been made, enhancing fertility in aging roosters remains a significant challenge.

**Methods:**

To identify the genes related to promoting sperm remodeling in aged Houdan roosters, we combined changes in testis and semen quality with transcriptome sequencing (RNA-seq) to analyze the synchrony of semen quality and testis development. In this study, 350-day-old Houdan breeder roosters were selected for RNA-seq analysis in testis tissues from induced molting roosters (D group) and non-induced molting roosters (47DG group). All analyses of differentially expressed genes (DEGs) and functional enrichment were performed. Finally, we selected six DEGs to verify the accuracy of the sequencing by qPCR.

**Results:**

Compared with the 47DG group, sperm motility (*P < 0.05*), sperm density (*P < 0.01*), and testis weight (*P < 0.05*) were significantly increased in roosters in the D group. Further RNA-seq analysis of the testis between the D group and 47DG group identified 61 DEGs, with 21 up-regulated and 40 down-regulated. Functional enrichment analysis showed that the DEGs were primarily enriched in the cytokine-cytokine receptor interaction, Wnt signaling pathway, MAPK signaling pathway, TGF-β signaling pathway, and focal adhesion pathway. The qRT-PCR results showed that the expression trend of these genes was consistent with the sequencing results. *WNT5A*, *FGFR3*, *AGTR2*, *TGFβ2*, *ROMO1*, and *SLC26A7* may play a role in testis development and spermatogenesis. This study provides fundamental data to enhance the reproductive value of aging roosters.

## Introduction

The reproductive performance of Breeder roosters plays a crucial role in poultry production. Breeder roosters experience a significant decline in semen quality as they age, putting them at risk of being culled in the subsequent years [1]. The fertility of roosters typically peaks at 12 months and then rapidly declines [2–5]. This decline was accompanied by a steady decrease in the quantity and quality of sperm per ejaculation [6, 7]. Aging is a highly intricate and irreversible process that takes place in all cells, tissues, and organs of living organisms [8]. It is one of the primary factors leading to the deterioration of various organs, including the reproductive system [9, 10]. Research has shown that even under better breeding conditions, aging still leads to a gradual loss of reproductive function and behavior in roosters [11, 12]. The testes are the primary reproductive organs in roosters responsible for sperm production and testosterone synthesis [13, 14]. There is ample evidence that the reduction in the activity of the gonadal axis associated with declining reproductive behavior is related to the aging phenomenon [15]. During the aging process, the morphological changes that occur in the testes are mainly characterized by a decrease in the volume and number of germ cells, as well as a decrease in semen volume and quality [5]. Roosters experience a decline in reproductive performance as they age, producing fewer viable sperm cells. This leads to a decrease in hatchability and significant economic losses in the poultry industry [13, 16, 17].

Molting is a natural and energy-demanding life history process of birds that helps them adapt to complex environmental changes [18]. Under natural conditions, hens begin to molt gradually after about 12 months of egg production [19]. Natural molting typically lasts for 3–4 months, and the molting process is not synchronized in the flock, resulting in lower egg production rates and inconsistent egg quality. This leads to a decrease in the overall productivity of the flock [20]. On the other hand, forced molting can be completed in about 4 weeks [21]. Forced molting is implemented by systematically restricting feed and implementing management protocols, which disrupt the hen’s natural metabolism and aging of the reproductive system [21]. Subsequently, providing water and feed again promotes cell regeneration and rapid development of reproductive organs, enabling the flock to complete molting within a short period and regain synchronous egg production, resulting in higher economic benefits [20]. As the measures implemented on laying hens are gradually lifted and the hens meet their energy requirements, their neural and endocrine functions are reactivated [22]. The survival potential of laying hens gradually recovers during this process [21]. Not only does the post-molt hen’s productivity significantly exceed that before molting [23], but the bacterial abundance in its feces is also more diverse [24], and its bone strength is stronger [25]. Numerous studies indicate that forced molting can prolong the lifespan of aged laying hens. Currently, forced molting of aging roosters to enhance their reproductive potential and extend their productive lifespan has not been tried.

With the development of sequencing technology, transcriptomes have been shown to have high accuracy in detecting differentially expressed genes (DEGs) related to important economic traits in chickens [21], They have been widely applied in the study of chicken growth and development [26, 27], reproductive traits [27, 28], and disease resistance [29]. Numerous studies have reported that light fasting or intermittent fasting was beneficial to the health of humans and animals, enhancing systemic immune function and slowing down aging [30, 31]. In chickens, study found that molting can stimulate testis growth and development in roosters [1]. However, the underlying molecular mechanism associated with the fasting-induced molting in roosters remains unclear, necessitating further investigation into the genetic networks responsible for this phenotype. Research into fasting-induced molting is gaining momentum. Our objective is to implement fasting-induced molting in Houdan breeder roosters at 350 days of age, aiming to enhance semen quality and, consequently, extend their economic life span. We plan to utilize transcriptome sequencing to identify DEGs subsequent to fasting-induced forced molting, thereby investigate its influence on the reproductive performance of the roosters.

## Results and analysis

### Effect of induced molting on semen quality and testis development of aged Houdan roosters

**Fig. 1:**
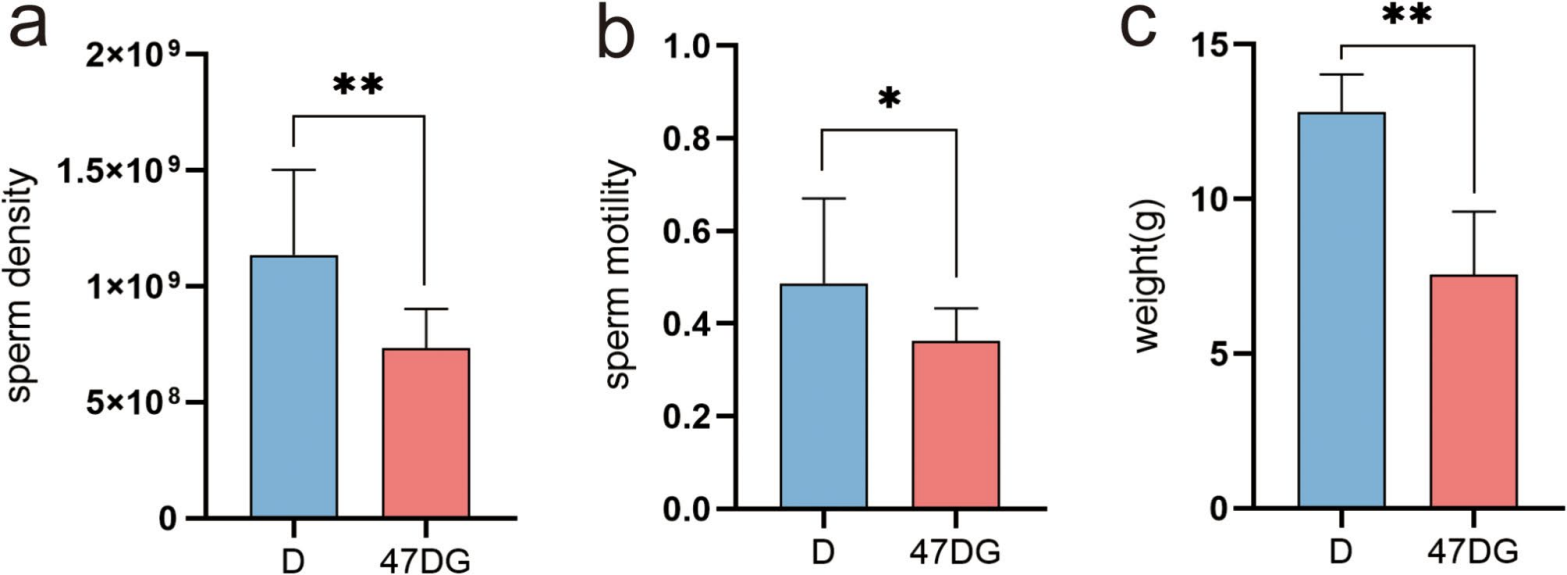
Effect of induced molting on semen quality and weight in testes of aged Houdan roosters. (**a**): Changes in sperm density. (**b**): Changes in sperm motility. (**c**): Changes in testis weight

To study the effects of induced molting on semen quality and testicular development in aged roosters, we statistically analyzed the changes in sperm vitality, sperm density, and testicular weight in the D group and the 47DG group of aged Houdan roosters. The results indicated that on the 32nd day of recovery following food restriction-induced molting, sperm density (Fig. 1a), the sperm motility (Fig. 1b), and testis weight (Fig. 1c) of aging Houdan roosters in the D group were all better than 47DG group. The difference in sperm vitality was significant (*P < 0.05*), and the differences in sperm density and testicular weight were highly significant (*P < 0.01*). These findings indicate that feed restriction-induced molt can improve testis weight, sperm motility, and sperm density in aging Houdan roosters.

### Transcriptome sequencing data analysis

The results of the transcriptome sequencing were shown in Table 1. Q20 was greater than 97%, Q30 was greater than 92%, and the percentage of GC content was above 50%. No separation phenomena were observed, indicating that the quality of the sequencing data was high and met the sequencing requirements, such that the next step could be carried out.

**Table 1:** Summary statistics of sequencing data

Sample name	Raw Date		Clean Date		Raw Q30 number	Q-sorce 20 (%)	Q-sorce 30 (%)	Overall alignment rate (%)	GC content (%)
	Raw reads	Raw bases	Clean reads	Clean bases					
47DG-1	40,462,974	6,109,909,074	38,294,600	5,782,484,600	5,692,664,543	97.53	93.17	92.74	52.1
47DG-2	42,213,232	6,374,198,032	39,944,522	6,031,622,822	5,965,299,542	97.67	93.58	92.61	51.5
47DG-3	41,083,774	6,203,649,874	38,870,434	5,869,435,534	5,733,607,200	97.18	92.42	92.53	51.54
D-1	41,542,392	6,272,901,192	39,261,158	5,928,434,858	5,881,719,805	97.73	93.76	92.70	51.29
D-2	42,556,110	6,425,972,610	40,298,350	6,085,050,850	6,009,313,144	97.67	93.51	92.39	52.56
D-3	42,798,076	6,462,509,476	40,485,520	6,113,313,520	6,020,299,449	97.5	93.15	92.44	51.8

*Notes* Q20, Q30: the percentage of bases with Phred values greater than 20 and 30 in the total number of bases, respectively

### Analysis of differentially expressed genes

**Fig. 2:**
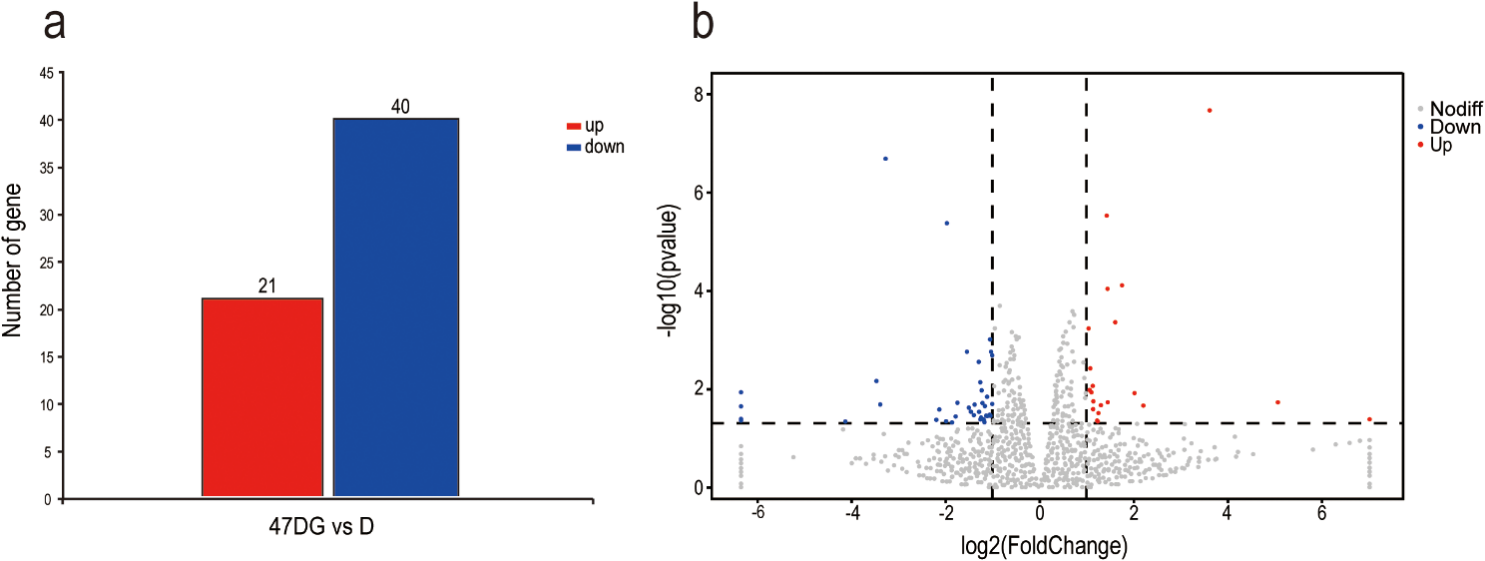
Differentially expressed genes in D group and 47DG group. (**a**): Results analysis of differentially expressed genes, (**b**): Volcano map of differentially expressed genes

The transcriptome sequencing data of the testis tissues of the D group and 47DG group on the 47th day were analyzed using DESeq2 software (Fig. 2). The results showed that compared with the 47DG group, the D group obtained a total of 61 significantly DEGs, including 21 significantly up-regulated genes and 40 significantly down-regulated genes.

### Genetic relationship between different group

**Fig. 3:**
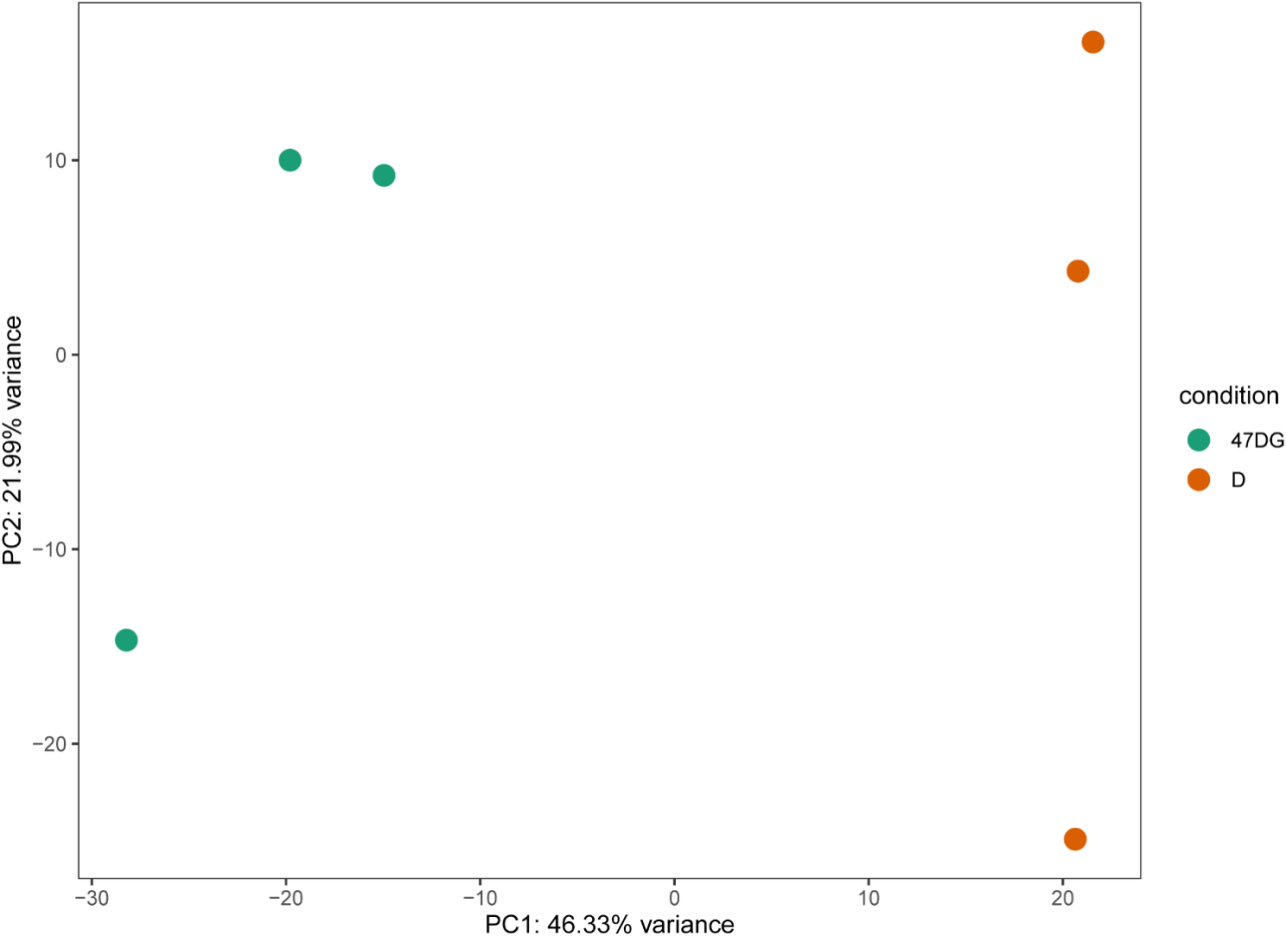
The principal component analysis (PCA) of the D group and 47DG group. main principal component (PC) scores as follows: PC1 = 46.33% and PC2 = 21.99%

**Fig. 4:**
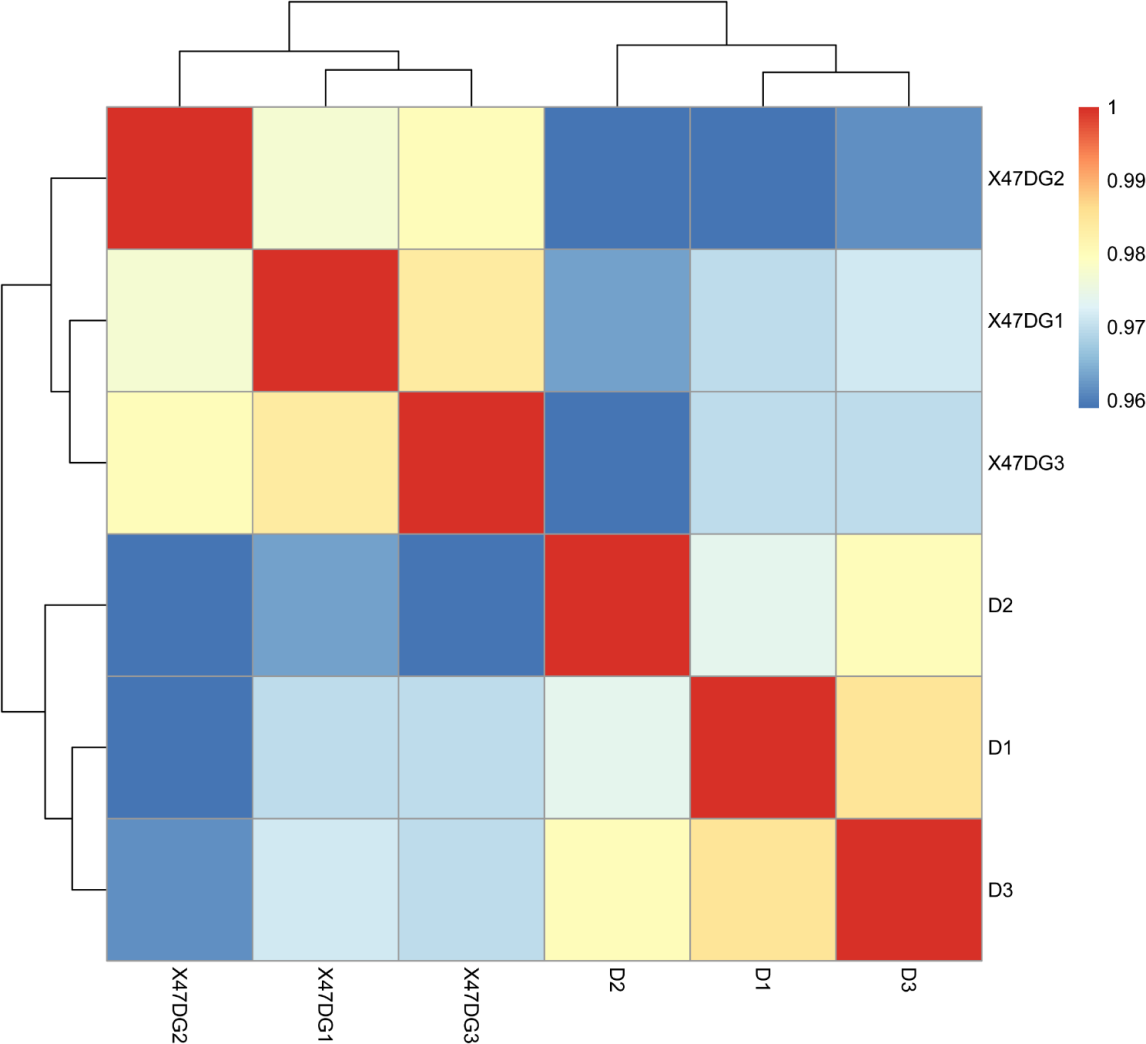
Heatmaps based on generated from six samples in the D group and 47DG group

The principal component analysis (PCA) plot showed that the D group and 47DG group were clearly separated with main principal component (PC) scores as follows: PC1 = 46.33% and PC2 = 21.99% (Fig. 3). Heatmaps based on generated from six samples in the D group and 47DG group. Results showed that were clearly separated (Fig. 4).

### GO and KEGG pathway enrichment analysis

GO functional enrichment analysis revealed that DEGs were enriched in three categories: biological process (BP), cellular component (CC), and molecular function (MF). A total of 518 significantly enriched GO terms were identified, including 443 in BP, 24 in CC, and 51 in- MF. As shown in Appendix 1, in the biological process category, DEGs were mainly involved in multicellular organism processes, positive regulation of response to stimulus, MAPK cascade regulation, extrinsic apoptotic signaling pathway, regulation of phosphorus metabolic process, cell adhesion, cell morphogenesis, cell growth, mesenchymal cell differentiation, and kidney system development, with 20, 10, 6, 3, 8, 7, 5, 4, 3, and 3 genes, respectively. In the molecular function category, DEGs were mainly involved in membrane, intrinsic component of membrane, extracellular region, plasma membrane, and vesicle, with 27, 20, 17, 16, and 8 genes, respectively. In the cellular component category, DEGs were mainly involved in signal receptor binding, receptor ligand activity, and growth factor activity, with 9, 5, and 2 genes, respectively.

**Fig. 5:**
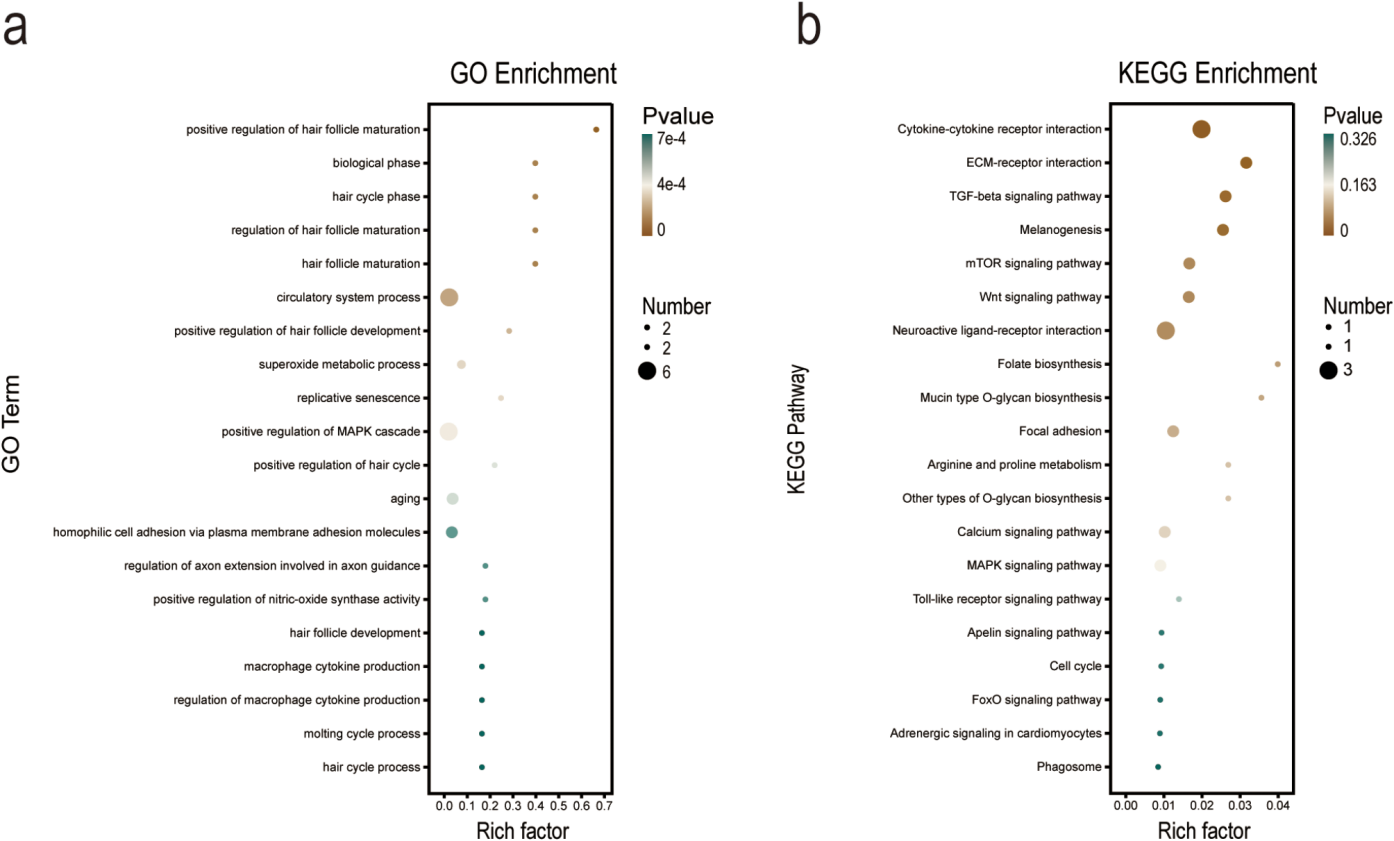
GO and KEGG pathway enrichment analysis. (**a**) Top 20 GO Term enrichment of DEGs, the bubble size represents the number of DEGs, and the bubble color represents the Q-value. (**b**) Top 20 KEGG pathway enrichment of DEGs, the bubble size represents the number of DEGs, and the bubble color represents the Q-value

KEGG pathway enrichment analysis revealed a total of 23 significantly enriched pathways. As shown in Fig. 5, the pathways significantly enriched in DEGs included cytokine-cytokine receptor interaction, neuroactive ligand-receptor interaction, ECM-receptor interaction, TGF-beta signaling pathway, melanogenesis, mTOR signaling pathway, Wnt signaling pathway, focal adhesion, calcium signaling pathway, and MAPK signaling pathway (Fig. 5).

### Verification of RNA-seq results using qPCR

**Fig. 6:**
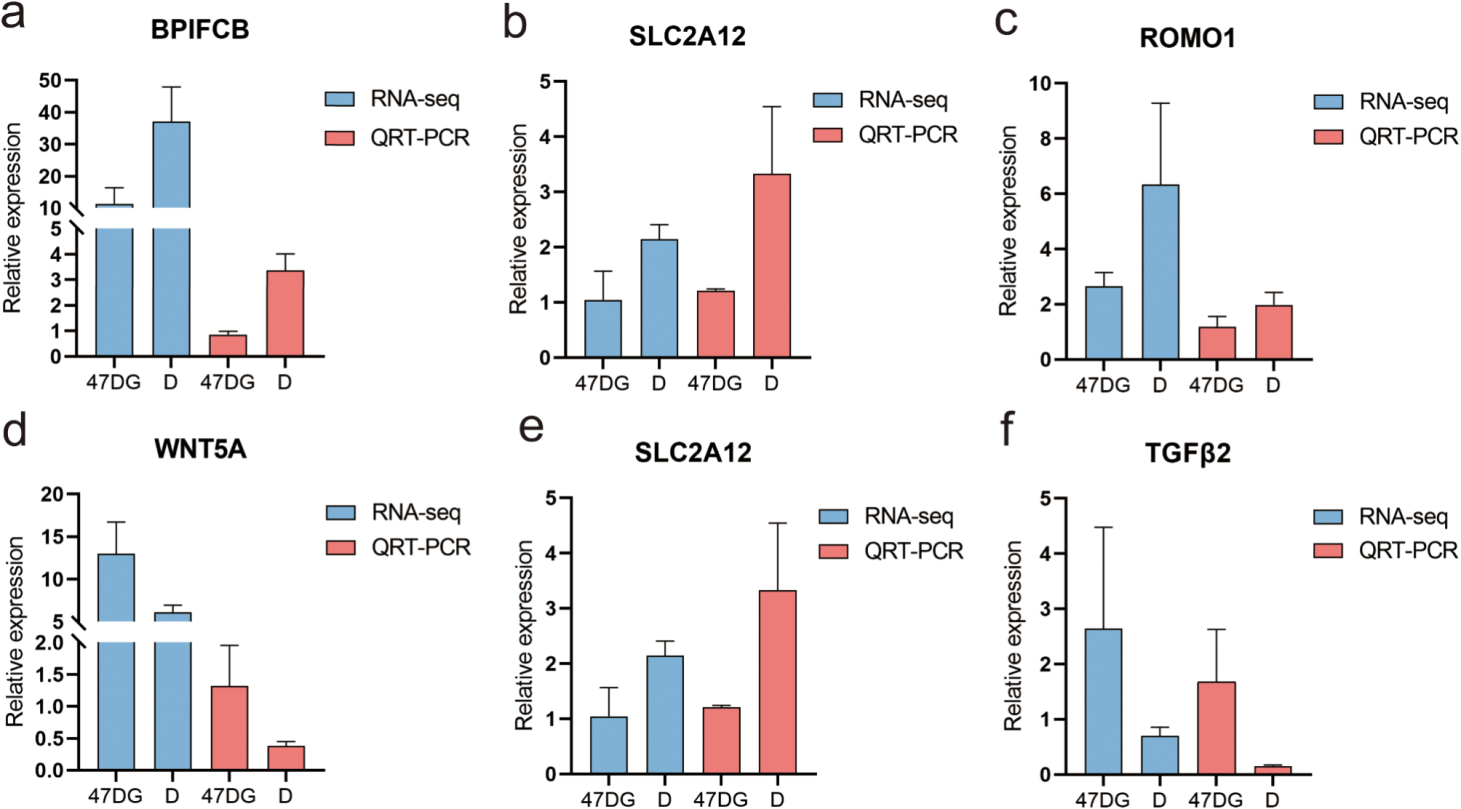
Expression comparison of 6 genes by qRT-PCR and RNA-Seq in group D and group DG. (**a**) BPIFCB; (**b**) SLC2A12; (**c**) ROMO1; (**d**) WNT5A; (**e**) SLC26A7; (**f**) TGFβ2

We performed qRT-PCR analysis the expression of 6 DEGs (*BPIFCB, SLC2A12, ROMO1, WNT5A, SLC26A7, TGFβ2*) (Fig. 6). Compared with 47DG group, the data from transcriptome sequencing showed that the 6 genes were significantly change in D group. The differential fold change was converted using log2(fold change), Our results showed that the expression profiles of the candidate unigenes revealed by the qRT-PCR data were consistent with those derived from transcriptome sequencing (Fig. 6), which indicated that the RNA-Seq analysis was reliable and provided a valuable gene sequence for biological analysis.

## Discussion

There is evidence to suggest a correlation between testis weight and semen quality capacity [1, 6]. Meanwhile, the testis is an important parameter for evaluating male reproductive capacity [32]. From our results, it can be perceived that the testis weight and semen quality of D Group roosters have been improved, which is consistent with previous research results [1]. In this study, 61 DEGs were identified by transcriptome sequencing of testis tissues from 350-day-old Houdan roosters, which underwent induced molting and recovery compared to those that did not undergo induced molting and recovery. GO functional and KEGG pathway enrichment analysis showed that these DEGs played important roles in the testis development and sperm generation process of roosters after induced molting. The DEGs were enriched in pathways related to cytokine-cytokine receptor interactions, neuroactive ligand-receptor interactions, ECM-receptor interactions, TGF-β signaling pathway, melanogenesis, mTOR signaling pathway, Wnt signaling pathway, focal adhesion, calcium signaling pathway, and MAPK signaling pathway. The DEGs detected in chicken testis tissue samples were mainly significantly enriched in steroid biosynthesis, lipid metabolism and glycerol lipid metabolism [33]. Li et al. found that cytokines and testosterone synergistically regulated the primary spermatocytes in sperm formation, sperm development, and the blood-testis barrier [34, 35]. Xiao et al. found that intercellular adhesion molecules had an antagonistic effect on the tight junction barrier of supporting cells, participated in the reconstruction of the supporting cell-blood-testis barrier, and regulated sperm adhesion in the epithelial cycle [36]. It was discovered by researchers Zhu et al. that the sperm motility and reproductive capacity of roosters were enhanced after induced molting. Through transcriptome sequencing analysis of rooster testicles, it was found that the regulation of cellular cytoskeleton is closely associated with spermatogenesis [1]. Cytokines play an important role in establishing and maintaining the immune characteristics of the testis [37, 38]. Testis cells produce various anti-inflammatory cytokines, which are important for the development of the testis and sperm generation.

The MAPK signaling pathway is one of the classical and important pathways in the eukaryotic signal transduction network. It is a key signaling pathway involved in cell proliferation, differentiation, apoptosis, and stress responses under normal and pathological conditions [39]. In this study, *FGFR3* and *TGFβ2* DEGs were significantly enriched in the MAPK signaling pathway in testis tissues. Several other studies [40–42] have shown that this pathway is involved in cell proliferation, differentiation, and apoptosis, making it an important signaling pathway. In mammals, MAPK can be translated as “mitogen-activated protein kinase” Regulating cell proliferation, differentiation, movement, and survival is one of the important pathways affecting testis development and spermatogenesis [43]. This suggests that the MAPK signaling pathway plays a critical role in regulating rooster testis development, spermatogenesis, and sperm quality. David M Ornitz et al. found that FGFR3 is a signal protein secreted by the FGF family, which mediates the intracellular signaling pathway with MAPK through the interaction between FGF ligands and their signal receptors [44]. FGF family members play a role in early embryonic development and organ development, and secreted FGF factors regulate basic cellular processes, including positive and negative regulation of proliferation, survival, migration, differentiation, and metabolism [44, 45]. FGFs also play an important role in adult tissues, often mediating metabolic functions, tissue repair, and regeneration by reactivating developmental signaling pathways [44, 46]. Elia Escasany et al. found that the transforming growth factor β (*TGFβ*) family of cytokines is a secreted multifunctional protein that is involved in cell proliferation and differentiation, wound healing, and fibrosis [46]. Study by Yang F et al. support the promoting role of TGF-β in neovascularization, which may be direct or indirect [47]. The Wnt signaling pathway plays an important role in development, regeneration, tumor development, and tissue repair [48]. The Wnt signaling pathway is usually divided into the canonical Wnt signaling pathway and the non-canonical Wnt signaling pathway, and Wnt ligands activate these two pathways by binding to different receptors on the cell surface [49]. The signals are transmitted to the cell nucleus through the Wnt and Ca^2+^ signaling pathways, activating downstream signaling molecules and regulating cell growth, morphology changes, and other processes [50]. *WNT5A* was typically involved in activating the non-canonical pathway and may inhibit the canonical pathway, thus participating in male germ cell development as a representative ligand involved in regulating male germ cell differentiation, and it was also a necessary ligand for the proliferation of testis cells [51]. In mammals, anti-Müllerian hormone (*AMH*) was produced by Sertoli cells that differentiate from the testis and by granulosa cells after birth [52]. Although *AMH* in birds is not identical to that in mammals [53, 54], it plays an important role in chicken gonadal development and maintaining testis morphology. This may also play an important role in the process of testis redevelopment and sperm production in roosters after fasting-induced molting in this study, but its specific functional role needs to be further explored.

Fibroblast growth factor receptor 3 (*FGFR3*), belonging to the fibroblast growth factor family, can specifically bind to the fibroblast growth factor receptor complex on the membrane of kidney and kidney tubular epithelial cells and regulate the reabsorption of calcium and phosphorus by activating the MAPK signaling pathway [36]. Ortiz-García CI et al. found that ROS produced by members of the NOX family play an important role in physiological processes such as sperm capacitation and acrosome reaction, and the ROS produced by NADPH oxidase (*NOX*) are associated with the activation of calcium-dependent proteinases and calmodulin-dependent proteinases [55]. Liu et al. found that ROS produced by NADPH oxidase can regulate the TGF-β signaling pathway through pathways including the MAPK pathway and the GTP-binding protein pathway [56]. Jankowska, A found that calcium-binding protein B (*S100B*) is present in most spermatogonia and sperm cells, and the Ca^2+^-regulated membrane-bound guanylate cyclase transduction mechanism plays a role in the biochemical action, molecular mechanism, and functional characteristics of spermatogenesis in the testis, mainly through *S100B* expression, transducing Ca^2+^-driven membrane-bound guanylate cyclase expression in germ cells [57]. This may be related to the significant improvement in sperm motility observed in this study. In summary, DEGs such as *WNT5A*, *FGFR3*, *AMH*, *TGFβ2*, *S100B* and *NOX4* may play important roles in the process of testis redevelopment in aging laying hens after fasting-induced molting.

## Conclusion

In this study, we performed induced molting experiment was conducted on 350-day-old Houdan roosters. Compared with the control group, the experimental group showed a significant increase in sperm motility, sperm density, and testis weight. Transcriptome sequencing analysis identified 61 DEGs, functional annotation revealed that genes such as *WNT5A*, *FGFR3*, *AMH*, *TGFβ2*, *S100B* and *NOX4* play important roles in testis development and spermatogenesis in elderly Houdan roosters after induced molting. This study provides basic data for further enhancing the breeding value of aging roosters and testis development after induced molting.

## Materials and methods

### Ethics statement

All the animal experiments were approved by the Animal Care Committee of the College of Animal Science and Technology, Henan Agricultural University, and were performed following the protocol approved by the Institutional Animal Care and Use Committee (IACUC) of China. All efforts were made to minimize animal suffering.

### Experimental animals and sample collection

In this experiment, seventy elderly aged Houdan roosters (fifty weeks of age) from the germplasm resources field of Henan Agricultural University were selected as experimental animals. They were divided into two groups: Group D and Group 47DG, with 35 roosters in each group. Group 47DG had free access to feed and water, while Group D underwent induced molting (Table 2: Feed Composition and Nutrient Content). The 47DG group was fed normally while the D group underwent induced molting (Table 2: Feed Coposition and Nutrient Content). The experiment lasted 47 days. The induction process included a 15-day fasting phase followed by a 32-day recovery phase. During the fasting phase, the roosters were deprived of food for 15 days, and water was withheld for 3 days (the first three days of the fasting period), with exposure to 8 h of light each day. During the recovery phase, the roosters began to gradually resume feeding starting from the 16th day of the experiment (30 g per day on days 16 and 17, 60 g per day on days 18 and 19, 90 g per day on days 20 and 21, and 120 g per day on days 22 and 23, after which they had free access to food and water), with an additional 0.5 h of light exposure added each day. On the 47th day of the experiment, three roosters from each group were randomly selected and euthanized by venous bloodletting after a 12-hour fast. Testicular tissues were promptly collected after slaughter, placed in RNA-free freezing tubes, and stored in liquid nitrogen. The testis transcriptome sequencing was outsourced to Nanjing Parsecno Gene Technology Co., Ltd.

**Table 2:** Feed Coposition and Nutrient Content

Raw	Cntent(%)	Ntrient Lvel	Cntent
Corn	65.0	Metabolizable Energy(MJ/Kg)	10.87
Soybean meal	23.0	Crude Protein (%)	15.50
Limestone powder	7.0	Lysine (%)	0.70
Pre-mixed feed	5.	Methionine (%)	0.32
		Methionine + Cysteine (%)	0.56
		Threonine (%)	0.50
		Calcium (%)	3.50
		Total Phosphorus (%)	0.60
		Available Phosphorus (%)	0.32
Ttal	100.0		

*Note* 1. Each kilogram of premixed feed provides Vitamin A 228,000 IU, Vitamin D 344,000 IU, 5-Hydroxyvitamin D3 138 µg, Vitamin E 1,500 IU, Vitamin K3 120.0 mg, Vitamin B1 60.0 mg, Vitamin B2 420.0 mg, Vitamin B6 120.0 mg, Vitamin B12 0.6 mg, D-Biotin 7.2 mg, D-Pantothenic Acid 360.0 mg, Folic Acid 60.0 mg, Niacin 1,200 mg, Choline Chloride 9,000 mg, Iron 1,600 mg, Copper 400.0 mg, Manganese 2,600 mg, Zinc 2,100 mg, Iodine 40.0 mg, Selenium 7.5 mg.2. The nutritional levels are calculated values

### Semen quality determination

The collected semen was diluted 500-fold with a 0.9% sodium chloride solution. A 10 µL mixed solution was taken, observed, and counted under a 40X microscope (Motic China) using a blood cell counting plate. Sperm counts were taken from the four corners and the central square of the counting chamber. (Sperm pressed against the boundary of the square were counted as left, not right, and up, not down.) The number of forward-moving sperm was counted to calculate the sperm density and sperm motility. All counting work was performed by the same person. Sperm density was calculated as the total number of sperm/80 × 500 × 400 × 10,000, and sperm motility (%) was calculated as the number of sperm with linear movement/total number of sperm × 100.

### The construction of cDNA library and sequencing

Total RNA was extracted from three left testis tissues of the experimental and control groups using Trizol reagent (Invitrogen Life Technologies, Shanghai, China). Afterward, the concentration, quality, and integrity were determined using a NanoDrop spectrophotometer (Thermo Fisher Scientific). After testing the samples, mRNA was purified using magnetic beads and oligo(dT) to bind the polyA tail of mRNA. Subsequently, the mRNA was fragmented using a fragmentation buffer. Single-stranded cDNA was generated using random hexamer primers from the mRNA template, and double-stranded cDNA was prepared using dNTPs and DNA polymerase I. The purified double-stranded cDNA underwent end repair, A-tailing, and adapter ligation, followed by size selection using AMPure XP beads. Subsequently, the final cDNA library was prepared by PCR amplification of the fragments. The sequencing library was then sequenced on the NovaSeq 6000 platform (Illumina, San Diego, CA) by Shanghai Personal Biotechnology Co. Ltd.

### Transcriptome sequencing data analysis and quality control

After sequencing, image files were obtained and converted using the sequencing platform’s built-in software to generate the original data in FASTQ format (raw data). Sequencing data contained a number of connectors and low-quality reads. Therefore, we utilized Cutadapt (v1.15) software to filter the sequencing data and obtain high-quality sequences (Clean Data) for subsequent analysis. All subsequent analyses were based on the clean data for high-quality analysis.

### Differentially expressed gene screening and functional enrichment

We used HTSeq (v0.9.1) statistics to compare the read count values for each gene as the baseline gene expression. The gene expression was normalized using FPKM (Fragments Per Kilobase per Million fragments). The differential expression of genes was analyzed using DESeq (v1.30.0) with the following criteria: a fold change in expression |log2FoldChange| > 1 and a significant P-value < 0.05. GO and KEGG pathway enrichment analyses were conducted using the topGO R package and the Orthology-Based Annotation System (KOBAS) software, respectively.

### Real-time PCR validation

To verify the accuracy of the transcriptome data, six DEGs screened during the experiment were randomly selected. The expression of testis differentially expressed genes was detected using qPCR with GAPDH used as the housekeeping gene. The primers for the target genes and internal reference gene (Table 3) were synthesized by Shangya Biotechnology Co., Ltd.The RT-qPCR protocol was as follows: one microgram of RNA was reverse transcribed to cDNA, and qPCR was performed in a 10 µL volume containing 1.0 µL of cDNA, 5.0 µL of SYBR Premix Ex Taq II (TaKaRa, Dalian, China), 0.5 µL of each primer (10 µmol/L), and 3 µL of RNase-free water. A LightCycler96 real-time PCR system (Roche Applied Science, Indianapolis, IN) was utilized. The amplification conditions were as follows: 94 °C for 5 min; 35 cycles of 94 °C for 30 s, 58 °C for 30 s, and 72 °C for 30 s; followed by a final 10-min extension at 72 °C. The relative expression of genes was calculated using the 2^−ΔΔCT^ method [58].

**Table 3:** List of primers used for qRT-PCR

gene	Primer sequence
BPIFCB	F: ACAGGAGCTTGGCCACTAAC R: TCATCAATCCGCACTGGGAC
SLC2A12	F: TATTGCTGAGATCGCCCCAC R: CCTGCAAGGCACCTAATGGA
ROMO1	F: CAGGATCGGCATGAGAGGAC R: TAGCCATGAACGTCCCGAAC
WNT5A	F: AGGAGCCCAGCCACTATGTA R: TGGCATTCCTTAATGCCGGT
SLC26A7	F: AGATGTCAAGGAGTGGTGCG R: ACACTGCATCAGGAACCAGG
TGFβ2	F: TACAGTGGGAAGACCCCACA R: GACGCAGGCAGCAATTATCC
GAPDH	F: GAACATCATCCCAGCGTCCA R: CGGCAGGTCAGGTCAACAAC

### Statistical analysis

Data were analyzed using one-way analysis of variance followed by Duncan’s multiple range test in SPSS version 23.0. P-values less than 0.05 were considered statistically significant. The data were presented as the average value ± standard error. GraphPad Prism version 8.0 was used for plotting.

## Data Availability

No datasets were generated or analysed during the current study.
